# Assessment of the Aerosol Generation and Toxicity of Carbon Nanotubes

**DOI:** 10.3390/nano4020439

**Published:** 2014-06-12

**Authors:** Patrick T. O’Shaughnessy, Andrea Adamcakova-Dodd, Ralph Altmaier, Peter S. Thorne

**Affiliations:** The University of Iowa, Department of Occupational and Environmental Health, 100 CPHB S320, Iowa City, IA 52242, USA; E-Mails: andrea-a-dodd@uiowa.edu (A.A.-D.); ralph-altmaier@uiowa.edu (R.A.); peter-thorne@uiowa.edu (P.S.T.)

**Keywords:** carbon nanotubes, aerosol generation, pulmonary response, inhalation, toxicity

## Abstract

Current interest in the pulmonary toxicity of carbon nanotubes (CNTs) has resulted in a need for an aerosol generation system that is capable of consistently producing a CNT aerosol at a desired concentration level. This two-part study was designed to: (1) assess the properties of a commercially-available aerosol generator when producing an aerosol from a purchased powder supply of double-walled carbon nanotubes (DWCNTs); and (2) assess the pulmonary sub-acute toxicity of DWCNTs in a murine model during a 5-day (4 h/day) whole-body exposure. The aerosol generator, consisting of a novel dustfeed mechanism and venturi ejector was determined to be capable of producing a DWCNT consistently over a 4 h exposure period at an average level of 10.8 mg/m^3^. The count median diameter was 121 nm with a geometric standard deviation of 2.04. The estimated deposited dose was 32 µg/mouse. The total number of cells in bronchoalveolar lavage (BAL) fluid was significantly (*p* < 0.01) increased in exposed mice compared to controls. Similarly, macrophages in BAL fluid were significantly elevated in exposed mice, but not neutrophils. All animals exposed to CNT and euthanized immediately after exposure had changes in the lung tissues showing acute inflammation and injury; however these pathological changes resolved two weeks after the exposure.

## 1. Introduction

This research group evaluated multiple aerosol generation methods for producing high, stable concentrations in a chamber designed for exposing mice to gases and aerosols [1]. Both wet and dry dispersion methods were tested. From the choices available, the majority of our subsequent inhalation toxicology experiments associated with the toxicity of metal and metal oxide nanoparticles relied on a wet method, the Collison nebulizer [2,3,4,5]. This generation method satisfied our criteria for a reliable aerosol generator defined as one that provided a consistent output over each exposure period and between exposure days. Preliminary trials while using the device allowed us to determine a nanopowder suspension concentration that resulted in a desired exposure aerosol concentration level. Further control was made through fractional adjustments to the suspension that resulted in similar fractional changes in the aerosol concentration.

The nebulizer generation method proved to be effective for producing a metal-oxide and metal nanoparticle aerosols [2,3,4,5]. However, a negative aspect of this method results from the production of nano-sized particles derived from the water used to create the suspension. Schmoll *et al*. provided particle size distribution plots resulting from an aerosol produced with water as well as plots produced from several suspension concentrations resulting from the addition of nano-TiO_2_ [1]. The plots clearly show an aerosol with a size mode near 20 nm resulting from the water-derived aerosol and a mode near 110 nm resulting from the TiO_2_ particles. Schmoll *et al*. found that the water-derived peak diminished with higher TiO_2_ suspensions, probably as a result of agglomeration of the water-derived particles to the TiO_2_ particles [1]. Although unreported, analysis of the water-derived particles by transmission electron microscopy/electron dispersive spectroscopy (TEM/EDS) suggest that they consist primarily of calcium and sodium salts resulting from these ions when the droplet evaporates that even exist in ultra-pure water such as Optima™ (Fisher Scientific, Pittsburgh, PA, USA) used for liquid chromatography. Others have reported similar results [6,7,8]. For example, Blackford and Simons utilized various grades of de-ionized and distilled water, as well as employing secondary filtration through small pore membrane filters, but were unable to eliminate a peak in nanoparticles derived from the nebulized water [6]. Given the presence of water-derived particles, our inhalation toxicology experiments were designed to compare the response induced by an aerosol created with a nanoparticle suspension to a control consisting of aerosolizing only water [3].

Our recent toxicological experiments have involved those that seek to better understand pulmonary responses to inhalation of carbon nanotubes (CNTs). Preliminary trials demonstrated that CNTs could be nebulized but the high suspension concentration needed to achieve high aerosol concentrations (in the mg/m^3^ range) enhanced nanoparticle agglomeration. To avoid this problem, as well as the occurrence of extraneous nanoparticles in an aerosol produced when nebulizing a water suspension, a dry generation system was investigated. We have successfully used a dry-powder aerosol generation system that relied on acoustic energy and a venturi aspirator to disperse aluminum oxide nanowhiskers [9]. However, multiple dry-dispersion methods were tested by Schmoll *et al*. to aerosolize carbon nanotubes (CNTs), including the acoustic generator, without success in terms of our reliability criteria [1].

Several papers describe dry-generating devices that produce a reliable CNT aerosol from a bulk powder [10,11,12,13]. However, these devices were either entirely custom-built and therefore difficult to accurately reproduce, or required a large amount of powder (>5 g) [11,13]. A vendor-supplied aerosol generator that showed promise for reliably producing a CNT aerosol was therefore purchased and tested prior to its use during a 5-day sub-acute study of CNT toxicity using a murine model. A primary objective of this study was therefore to characterize a dry-dispersion aerosol generator when producing a CNT aerosol. The factors that affect aerosol generation were also tested with regard to desirable properties of an aerosol generator used for inhalation toxicology experiments involving CNTs. As described by Schmoll *et al*., those properties include: (a) a consistent concentration over many hours at a desired level; (b) an aerosol composition free from contaminants; and (c) a consistent aerosol size distribution over time that has both a small geometric mean diameter (µ_g_ < 200 nm) and small geometric standard deviation (σ_g_ < 2.5) [1].

## 2. Methods

### 2.1. Aerosol Generator

**Figure 1:**
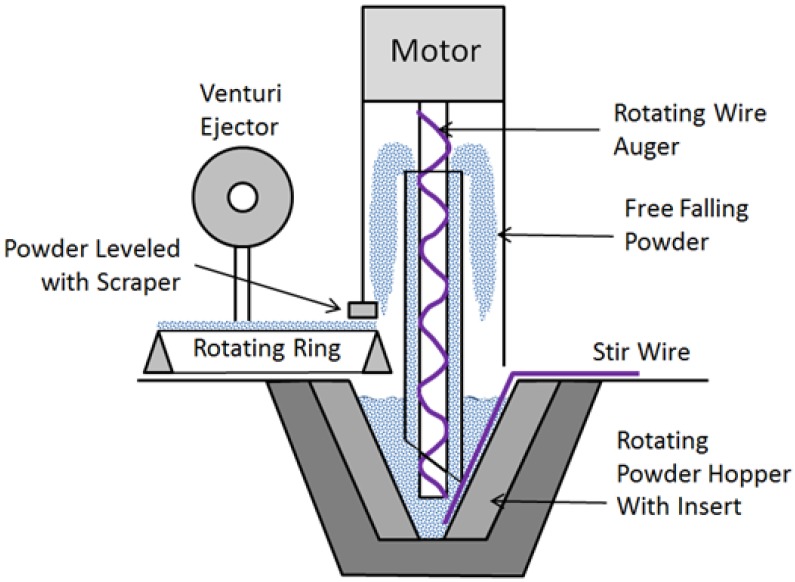
Dust generator schematic diagram (not to scale).

An aerosol generator (Model SAG 410/U, TOPAS GmbH, Dresden, Germany) was purchased in 2010 as an option for dispersing aerosols from a bulk powder in the dry state. The generator incorporates both a dispersing unit and a dust feeder. The disperser is a venturi aspirator that operates at flow rates between 25 L min^−1^ and 67 L min^−1^. The dust feeder consists of multiple parts that combine to deliver powder to the suction nozzle of the venturi (Figure 1). Powder is placed in a conical, rotating hopper. A rotating auger, consisting of wire wrapped loosely around a rod, pulls powder up through a tube. To enhance the auger’s ability to pick up powder, a wire extends straight down the side of the hopper wall to agitate powder as it moves past the wire. Powder that reaches the top of the tube forms a cloud of falling particles within a column. As shown in Figure 1, the falling particles land on a rotating ring protruding into the column space. The particles settling on the upper edge of the ring are then scraped to an even height prior to exiting the column and carried to the suction nozzle of the venturi ejector to be aerosolized.

**Figure 2:**
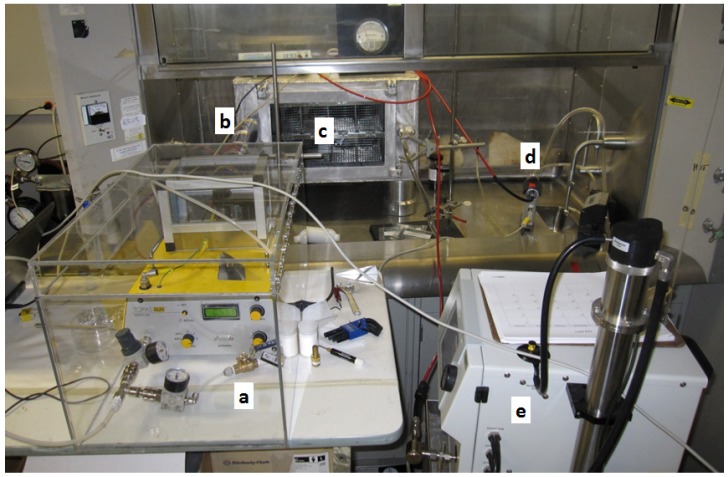
Aerosol delivery system consisting of the (**a**) aerosol generator in an acrylic plastic enclosure; (**b**) charge neutralizer; (**c**) whole body exposure chamber; (**d**) aerosol photometer; and (**e**) scanning mobility particle sizer.

As supplied by the manufacturer, the powder hopper had a total volume of approximately 350 cm^3^ but powder could only be added to half the height to prevent spill-over, and with the auger tube, resulted in an effective volume of powder of 135 cm^3^. As shown in Figure 1, an aluminum insert was fabricated to reduce the effective powder volume to 30 cm^3^. This alteration allowed as little as 0.5 g of powder to be added to the hopper and still surround the auger to a depth sufficient to cause it to be consistently pulled up by the auger. No other adjustments were made to the purchased unit. A glass box was supplied by the manufacturer to surround the feeder and ejector. Since the ejector pulls in air along with particles on the ring edge, HEPA filtered air is supplied to the space within the glass box. We also built a polypropylene box large enough to house the entire generator and with a connection to laboratory vacuum air to maintain a negative pressure relative to room air to minimize the possibility of aerosol emission into the laboratory (Figure 2).

### 2.2. Generator Characterization Trials

To characterize the performance of the generator for aerosolizing a CNT aerosol, double walled carbon nanotubes (DWCNTs) were purchased (Stock No. 1290NMG, Nanostructured & Amorphous Materials, Houston, TX, USA). The DWCNTs consisted of >90% carbon nanotubes of all types (single-walled and multiwalled) and >50% double-walled carbon nanotubes. As reported by the manufacturer, the outer diameter of these DWCNTs was < 5 nm, the inner diameter ranged from 1.3 to 2.0 nm, and with a length 5–15 μm and surface area ~400 m^2^/g. Additional characterization included the use of transmission electron microscopy (TEM) to size outer tube diameters, which ranged between 1 nm and 6 nm with an average of 3.3 nm. BET surface area analysis was also performed which resulted in a higher value of 575 m^2^/g than that reported by the manufacturer. The bulk powder was also analyzed for metals that can often appear as contaminants in DWCNTs. The University of Iowa State Hygienic Laboratory performed a qualitative screen for 21 metals following a modified EPA method 3050B (200.8), using inductively coupled plasma-mass spectroscopy (ICP-MS). No detectable level (>2 µg/g) of any metal was obtained.

The primary variables that affect the generation rate include the width of the top edge of the rotating ring; the rotation speed of the ring, and the height of powder on the ring. Two rings are provided: one with a top edge of 0.3 mm and the other with a top edge of 1 mm. The bulk characteristics of the powder may also influence the generation rate, but this study was restricted to the use of the generator for aerosolizing one type of carbon nanotubes. Factors that may not affect the generation rate but could influence the consistent dispersion of powder include the rotation speed of the auger wire, the depth of the powder in the hopper, and the air pressure to the venturi ejector.

The DWCNT bulk powder was placed in a drying oven at 100 °C overnight prior to its use in the aerosol generator. To test the effect of changes in the operational variables of the generator, the generated aerosol flowed into a small, 65 L, aluminum chamber (Figure 2) used for mouse whole-body exposures [14]. Aerosol exiting the generator first passed through a charge neutralizer (Model 3012, TSI Inc., Shoreview, MN, USA) before entering the chamber to follow our standard procedure for a mouse exposure. A vacuum pump on the exhaust side of the chamber was used to pull air at a slightly higher rate than provided by the generator to achieve a total flow of 32 L min^−1^ and a negative static pressure of −10 mm H_2_O.

The size distribution of the DWCNT aerosol was measured primarily with a scanning mobility particle sizer (SMPS, TSI Inc., Shoreview, MN, USA) set to size and count particles in 105 size channels between 7 nm and 289 nm. To verify that the entire particle size distribution was measured, a portable optical particle counter (Model 1.108, Grimm Technologies, Inc., Douglasville, GA, USA) was also used to provide counts in 15 size channels between 300 nm and 20,000 nm. Inlet hoses to these instruments were attached to a Y-connector that terminated at a port placed immediately above the exhaust-air port of the chamber. DWCNT mass concentration was determined from gravimetric analysis of filter samples taken by placing a 37-mm filter in a filter holder placed in-line with the chamber exhaust air. Filters were pre- and post-weighed in a dedicated, climate-controlled room housing a six-place microbalance (Model XP26, Mettler-Toledo, Inc., Columbus, OH, USA).

Generator characterization trials consisted of measuring changes in chamber aerosol concentration after step-changes in ring speed. Those measurements were made with an aerosol photometer (Model pDR-1200 RAM, Thermo-Electron Corp., Waltham, MA, USA) set to take readings every 1 s. The relationship between photometer readings and filter-based concentrations was also analyzed to determine whether that instrument could be employed as the sensor in an aerosol concentration feedback control system utilizing signals to the generator to vary ring speed. Measurements to establish the consistency of aerosol concentration over hour-long periods were also performed as part of a study to characterize the sub-acute toxicity of DWCNT using a murine model.

## 3. Toxicity Study Methods

### 3.1. Animals

Male mice (C57Bl/6, The Jackson Laboratory, Bar Harbor, ME, USA) were used in this study. Before the beginning of the exposures, mice were acclimatized for 12 days in vivarium in polypropylene, fiber-covered cages in HEPA-filtered Thoren caging units (Hazelton, PA, USA). Animals were provided with food (sterile Teklad 5% stock diet, Harlan, Madison, WI, USA) and water (via an automated watering system) *ad libitum.* Regular light-dark cycle (12 h) was maintained in the animal room. This study protocol was approved by the Institutional Animal Care and Use Committee at the University of Iowa. Mice were exposed in the whole-body chamber for 4 h/day for total of 5 days and necropsied either within one h (0 week) or 2 weeks after the last exposure (2 weeks). Sentinels animals (with no exposure) were used as controls. Five animals were used for each exposed group and three were used for controls.

### 3.2. Bronchoalveolar Lavage Fluid

The overdose of isoflurane followed by cervical dislocation and exsanguination through the heart was used to euthanize the animals at 0 week and 2 weeks post exposure. Bronchoalveolar lavage (BAL) was performed *in situ* using a total of 3 mL (3 times 1 mL) of 0.9% sterile sodium chloride solution (Baxter, Deerfield, IL, USA). Recovered cells from BAL fluid were then processed for enumeration of total cells using a hemocytometer. The remaining BAL cells were fixed on microslides using Cytospin 4 (Thermo Shandon, Thermo Scientific, Waltham, MA, USA), stained with hematoxylin and eosin and number of macrophages, lymphocytes, netruophils and eosinophils was counted using a light microscope (total of 400 cells/each sample).

### 3.3. Histopathology of Lung Tissues

Lungs (*n* = 3 from each CNT-exposed group and 2 controls) for histopathology evaluation were not lavaged but fixed in 10% buffered formalin via the cannulated trachea. The tissues were then paraffin-embedded, sectioned at 5 μm, and stained with hematoxylin and eosin (H & E) and evaluated by a board-certified veterinary pathologist using light microscopy with the focus on parenchymal architecture (bronchioles, alveoli, pleura, and vasculature), and presence or absence of inflammatory cell infiltrates, evidence of acute lung injury, lymphoid agglomerates or fibrosis.

### 3.4. Statistical Analyses

Measured outcome parameters from mice exposed to CNT aerosol and controls were compared using *t*-tests for equal or unequal variances or one-way analyses of variance (ANOVA) (SAS Ver. 9.2, SAS, Inc., Cary, NC, USA). A *p*-value less than 0.05 was considered statistically significant. Data are expressed as mean ± standard error (SE) unless otherwise noted.

## 4. Results and Discussion

### 4.1. Initial Generator Performance Characteristics

Initial trials (unreported) demonstrated that the fabricated hopper insert did not alter the intended actions of the generator to produce an aerosol. The stir wire was bent to accommodate the sides of the hopper with the insert. Use of the wire was essential for maintaining a steady feed of powder onto the rotating ring. The bottom hole of the tube through which material is lifted by the auger has a 45° taper as shown in Figure 1. We found that the taper should be rotated away from the wire to maximize the lift of powder by the auger.

The rotation speed of the auger and hopper are controlled by the same motor. Initial trials demonstrated that a rotation speed at least 50% of maximum was needed to allow the auger to pick up and carry DWCNT to the top of its length to initiate the dustfeed process to the rotating ring below. Further increases in auger rotation speed did not noticeably increase aerosol generation rate. It is likely that higher auger speeds carried more DWCNT but the limiting factor is the amount that can settle onto the top edge of the rotating ring, therefore additional amounts of DWCNT settling onto the ring did not increase the amount carried to the venturi ejector. An attempt was also made to use the scraper to provide a consistent height of DWCNT settled onto the ring, but this created gaps in the settled particles as some were intermittently pushed off by the scraper. Therefore, the scraper was raised so as not to have an effect on the ring powder height.

### 4.2. Generator Transient Characteristics

**Figure 3:**
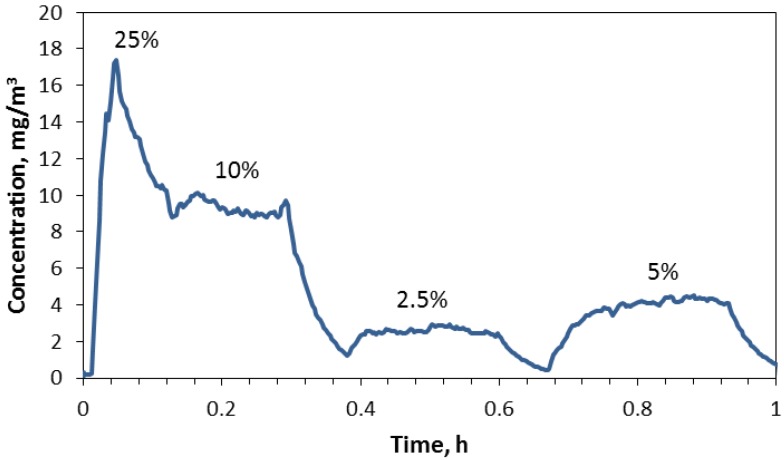
Changes in chamber concentration with step changes in ring speed when using the 0.3 mm ring.

Several trials were performed to test the responsiveness of the aerosol produced relative to the rotation speed of the ring; a typical plot of concentration over time measured with the aerosol photometer is given in Figure 3. The rotation speed was originally set at 25% of maximum rpm to obtain a relatively high concentration (>10 mg/m^3^) followed by step changes to 10%, 2.5% and 5% of maximum rpm. The fast response after a change in speed is a consequence of the system used which operated with a relatively high air exchange rate of 30 h^−1^. The DWCNT concentration remained relatively stable after the transient response caused by a change in ring speed.

The reason for dips in concentration after either an increase or decrease was not determined but suggests a temporary slowdown in the ring speed. As we noted in other trials, the average concentration was not precisely associated with the relative difference in ring rotation speed. For example, changing the ring speed from 10% to 2.5% of maximum rpm reduced the concentration by 28% rather than 25%, and increasing from 2.5% to 5% of maximum rpm increased the concentration by 158% rather than 200%. Other trials produced similar response patterns and steady concentrations after a change that increased or decreased as expected but were not precise relative to the ratio of ring speeds induced in the trial.

To test the consistency of the generator between trials, the 1-mm ring was attached and the system started and allowed to reach a steady state with a ring rpm of 2% of maximum at which time a filter measurement was made for at least 10 min. The average of 10 of these trials was 13.9 mg/m^3^ with a range of 11.3–16.2 mg/m^3^, or an absolute percent difference of 19% and 14%, respectively.

Based on the width of the top edge of the two rings, 0.3 and 1.0 mm, and assuming the powder forms a semicircular cylinder on the top of the ring when the scraper is not used, then the 1.0 ring should carry a volume of powder that is 11 times greater than the 0.3 ring. The slope of concentrations relative to ring speeds when using the 1-mm ring was 6.15 when the intercept was forced to 0. Likewise, the slope when using the 0.3 mm ring was 0.88. Dividing these slopes produced a ratio of 7.0 indicating that the mound of powder on the ring edge is not perfectly semicircular, and it was likely that the powder height varied.

### 4.3. Generator Steady State Characteristics

**Figure 4:**
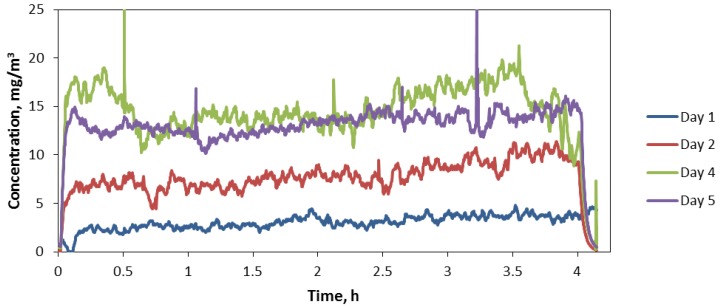
Aerosol concentration time series during mouse exposure days (excluding Day 3).

Average daily concentrations obtained gravimetrically were 1.5, 7.5, 17.6, 14.2, and 13.4 mg/m^3^ over the 5-day exposure period, respectively, with an overall average concentration of 10.8 mg/m^3^ and standard deviation of 6.4 mg/m^3^. Aerosol concentrations over each of the 4 h daily exposure period were relatively stable (Figure 4). Figure 4 excludes concentrations recorded during Day 3 when the ring speed was purposely altered to optimize the concentration level near 10 mg/m^3^. As shown in Figure 4 concentrations remained low during day 1 and increased on subsequent days. A reason for not maintaining concentrations near our target concentration early in the exposure was the lack of previous long-term use of the generator and the high output rate of the generator. The high output rate necessitated setting the ring speed near its lowest value, 2%, which did not allow regulating concentrations to lower levels. Furthermore, we determined that the 45° opening at the bottom of the auger tube was facing an area of the hopper beyond the stir wire, an area with a stagnant zone of powder. Turning the opening to face the area just in front of the wire where powder is most agitated significantly increased the auger’s ability to capture and lift powder up the tube.

**Figure 5:**
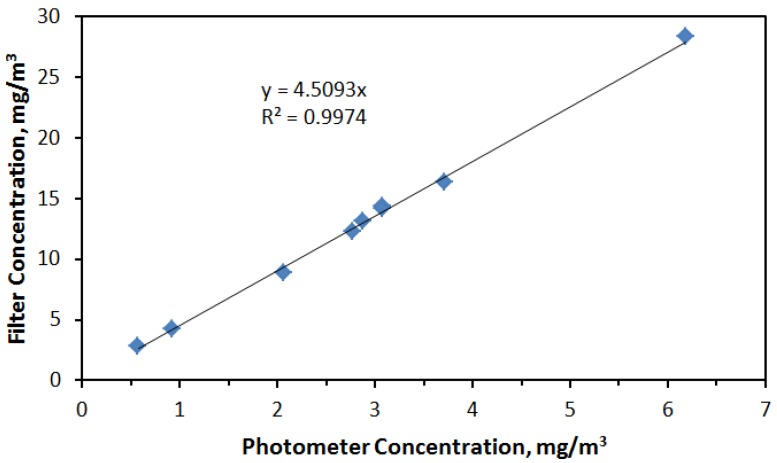
Linear relationship between photometer readings and associated aerosol concentrations measured gravimetrically.

The concentrations shown in Figure 4 were adjusted from those measured with the aerosol photometer based after relating filter concentrations with the average of photometer readings taken over the same time period that a filter measurement was made. As shown in Figure 5, the relationship between average photometer concentration and corresponding filter measurement was highly linear resulting in the relationship: photometer reading × 4.5 = actual concentration.

**Figure 6:**
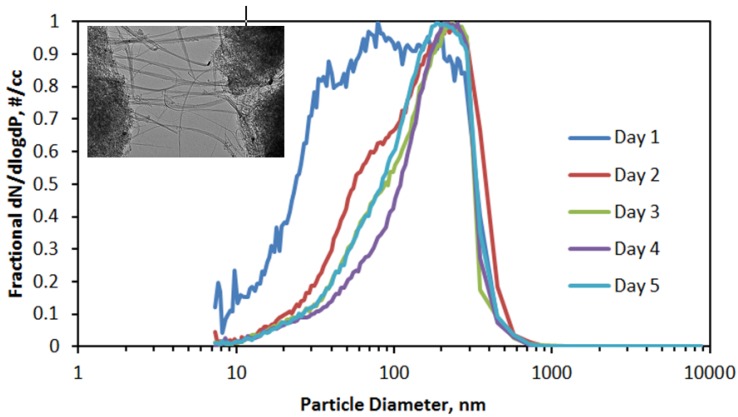
Average of aerosol size distributions measured during 4 h exposure periods (counts normalized to the maximum count), with inset of transmission electron micrograph of double-walled carbon nanotubes (DWCNT) fibers emanating from a fiber bundle.

The size distributions of the aerosol produced each day are shown in Figure 6. Other than for Day 1, when concentrations were very low, the aerosol size distribution remained constant over the other four exposure days. The overall average count median diameter (CMD) was 121 nm, and the average geometric standard deviation (GSD) was 2.04. From those values, a mass median diameter (MMD) of 548 nm was calculated using the Hatch-Choate conversion equation [15].

During each trial, the mass of DWCNT powder added to the hopper was weighed and then reweighed after the 4 h exposure. The aerosol generation rate resulting from this procedure averaged 1.5 mg/min (0.09 g/h). Given that these trials at a ring speed near 2% the estimated 100% ring speed generation rate is 76 mg/min (4.6 g/h), which is comparable to rates reported by the manufacturer for other dust types when considering the low bulk density of carbon nanotubes (0.12–0.14 g/cm^3^). Although not employed in this study, the application of feedback control is possible by way of electrical connections on the generator body that can receive a voltage signal to automatically vary ring speed relative to a desired concentration. Control schemes could be applied to filter the stochastic aerosol concentration signal from a photometer in the chamber such as that described by O’Shaughnessy and Haugh [16] or to apply a proportional-integral-derivative (PID) scheme directly to the unfiltered signal as described by McKinney *et al*. [11].

### 4.4. DWCNT Toxicity Study

#### 4.4.1. Deposited Dose

We estimated that total airway deposited dose was 32 µg per mouse (1.9 mg/kg). This estimation was based on an average body weight of 25 g, breathing frequency of 165 breaths/min, tidal volume of 0.15 mL/breath, aerosol concentration in the whole-body chamber of 10.8 mg/m^3^, the duration of exposure of 20 h and particle deposition fraction (α) in total airway of 0.10. The deposition fraction was computed using the Multiple-Path Particle Dosimetry Model (MPPD Ver. 2.11, Applied Research Associates, Raleigh, NC, USA) based on particles with a median diameter of 100 nm and density of DWCNT of 0.15 g/cm^3^ [17].

#### 4.4.2. BAL Cells

**Figure 7:**
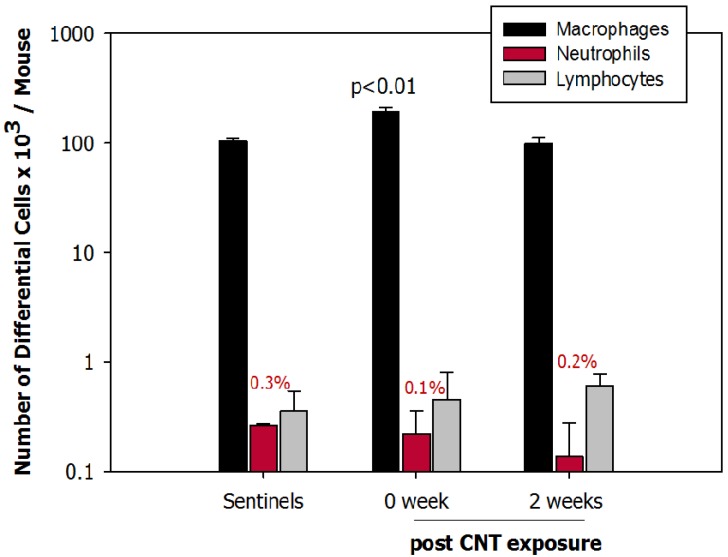
Number of macrophages, neutrophils and lymphocytes in bronchoalveolar lavage (BAL) fluid in controls (sentinels), animals exposed to carbon nanotube (CNT) euthanized immediately (0 week) or 2 weeks post exposure (2 weeks).

The total number of cells in BAL fluid was significantly (*p* < 0.01) increased in mice exposed to DWCNTs for 4 h/day for 5 days (192 × 10^3^ cells/mouse) compared to controls (105 × 10^3^ cells/mouse). Similarly, macrophages in BAL fluid were significantly elevated in exposed mice (*p* < 0.01). The number of macrophages at 2 weeks post exposure returned to baseline data. The number of neutrophils (a sign of an inflammatory response) in mice necropsied immediately (0 week) or 2 weeks post exposure was not significantly different from controls (Figure 7). No eosinophils were found.

**Figure 8:**
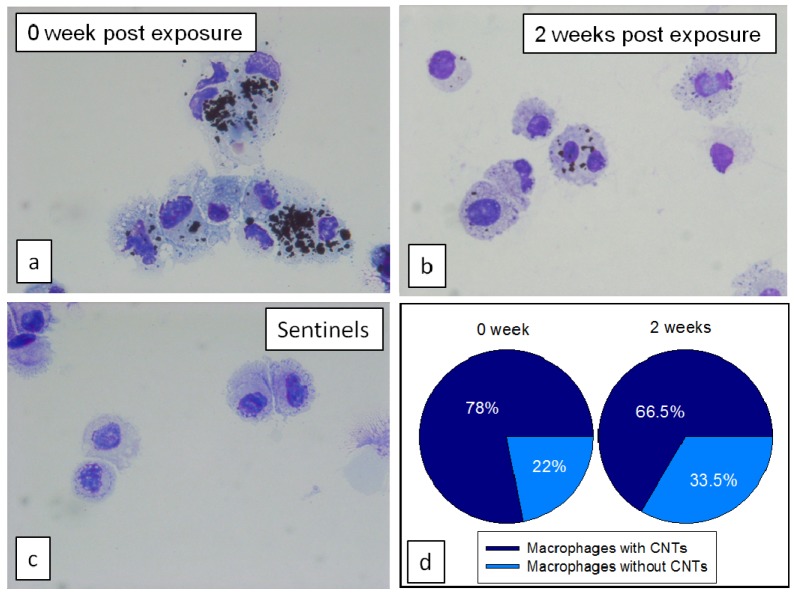
BAL macrophages from mice necropsied (**a**) immediately; or (**b**) 2 weeks after last exposure to CNTs; and (**c**) from control mice without exposure; (**d**) Pie charts represent percentage of macrophages population with and without CNTs.

The percentage of particle-laden macrophages in BAL fluid as well as number of particles engulfed in macrophages at 2 weeks post exposure was lower than at 0 week, however majority of macrophages still contained some CNTs (Figure 8). Immediately after exposure, there was 78% particle-laden macrophages, at 2 weeks post exposure this number decreased only slightly to 66%, however the number of engulfed particle agglomerates in macrophages was much lower as shown in Figure 8b.

#### 4.4.3. Lung Histopathology

**Figure 9:**
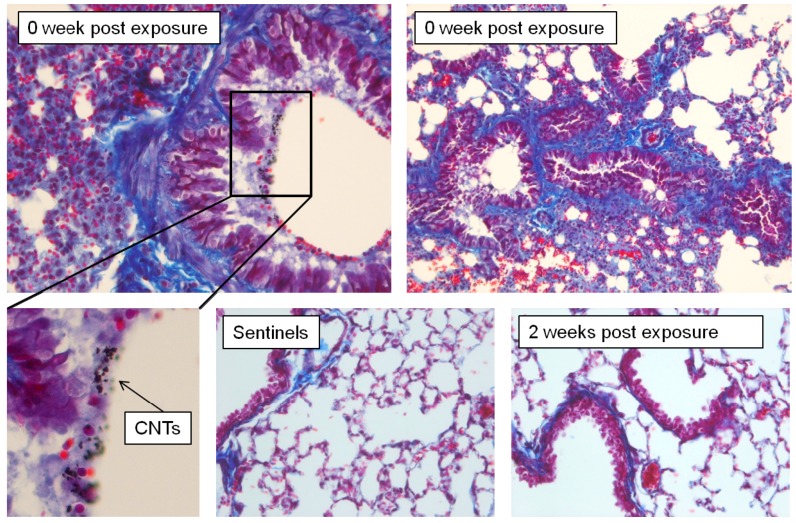
Mice exposed to CNT and necropsied at 0 week post exposure developed acute inflammation/injury. Lungs were partially atelectatic with coalescing vascular congestion. Overt fibrosis characterized by fibroplasia and collagen deposition was not detected in these mice.

All animals exposed to CNT and euthanized at 0 week post exposure had changes in the lung tissues showing acute inflammation and injury. The inflammation was found primarily in the perivascular peribronchial regions as uncommon loose aggregates of mononuclear cells (macrophages and lymphocytes). The epithelium of larger airways appeared to be hyperplastic with some goblet cells change. Some platelet plugs were seen in many vessels (platelet plugs are formed if blood vessel wall is damaged and collagen is exposed after vessel injury). Explicit fibrosis characterized by fibroplasias and collagen deposition was not detected. Such changes can often be seen also in control animals following euthanasia procedures. Enhanced collagen blue staining around airways and vessels might be an exaggerated artifact due to atelectasis present in these samples (Figure 9). All pathological changes were resolved in 2 weeks post exposure. The lung tissues from controls and animals euthanized 2 weeks post exposure did not show major pathological changes.

Our results are contrary to Sager *et al*. 2013 [18]. In that study the exposure of C57Bl/6 mice to DWCNTs in suspension by pharyngeal aspiration (40 μg/mouse) caused a significant pulmonary inflammation represented by an increased number of neutrophils in BAL fluid. Another study also showed a pulmonary neutrophilic inflammation in BAL fluid after intra-tracheal instillation of functionalized (block copolymer dispersed) DWCNTs to mice (50 μg/mouse) [19]. A study with a similar dose (40 μg/mouse) of single-walled carbon nanotubes (SWCNTs) by pharyngeal aspiration found robust but acute inflammation [20]. The mice also developed early onset of progressive fibrosis and granulomas. These findings further confirm that the exposure method plays an important role in pulmonary responses as has been shown in a comparative study of pathological changes induced by multiwalled carbon nanotubes (MWCNTs) after intra-tracheal instillation *versus* inhalation [21] as well as in the case of other nanomaterials such as TiO_2_ [3]. Our estimated dose of 32 μg/mouse was within the range mentioned by two other studies [18,19]. However, this dose was delivered over a period of 5 days, and represented the physiological and active process of inhaling respirable particles, as opposed to delivering a bolus dose of larger agglomerates as in the case of exposure to particulates in suspension. Moreover, inhalation exposure mimics more closely the exposure that might occur in occupational or environmental settings.

To our surprise, we found that DWCNTs in our study were clearing from the lungs by macrophages, the lungs at 2 weeks post exposure were not different from sentinels. This was also demonstrated by a lower number of CNT agglomerates in macrophages obtained by BAL at 2 weeks post exposure.

It is important to mention that the toxicity part of this study was not designed for a full assessment of pulmonary toxicity of DWCNTs but rather as a proof of concept of successful implementation of a dry-powder generation system for evaluation of potential toxicity of any type of CNTs after inhalation. Nevertheless, our limited assessment of pulmonary toxicity after inhalation of DWCNTs showed interesting results and more studies focused on a wider range of toxicity biomarkers are warranted.

## 5. Conclusions

A commercially-available aerosol generator was shown to reliably produce a DWCNT aerosol directly from the dry powder. The novel dust-feed mechanism proved to be capable of continuously supplying a consistent amount of DWCNT powder to a venturi ejector. The resulting chamber concentration could be easily manipulated by varying the speed of the ring carrying powder to the ejector. Average concentrations over a 4 h exposure period remained relatively stable. The method of generation of CNTs for inhalation toxicity assessment studies plays a major role in a potential pulmonary toxicity. The results of our study show that inhalation exposure to DWCNTs at a relatively high estimated dose of 32 μg/mouse caused an acute inflammation and injury in the lung tissue, but these pathological changes resolved 2 weeks after the exposure.
